# Soil and Foliar Applications of Wood Distillate Differently Affect Soil Properties and Field Bean Traits in Preliminary Field Tests

**DOI:** 10.3390/plants12010121

**Published:** 2022-12-26

**Authors:** Michelangelo Becagli, Iduna Arduini, Valentina Cantini, Roberto Cardelli

**Affiliations:** Department of Agriculture, Food and Environment, University of Pisa, 56124 Pisa, Italy

**Keywords:** flower fertility, nitrate, nodules, phosphorus, soil enzymes, *Vicia faba* var. *minor* beck, wood vinegar

## Abstract

Natural products such as wood distillate (WD) are promising alternatives to xenobiotic products in conventional agriculture and are necessary in organic farming. A field study gave insight into the effectiveness of WD applied as foliar spray (F-WD), soil irrigation (S-WD), and their combination as growth promoters for field beans. The soil fertility and quality parameters, plant growth, nutrient uptake, and resource partitioning within plants were evaluated. In a pot trial, we tested the effect of S-WD on root nodule initiation and growth. S-WD increased DOC and microbial biomass by approximately 10%, prompted enzyme activities, and increased nitrate and available phosphorus in soil, without affecting the number and growth of nodules in field beans. In contrast, the F-WD slightly reduced the DOC, exerted a lower stimulation on soil enzymes, and lowered the soil effect in the combined distribution. In field beans, the F-WD reduced the stem height but increased the number of pods per stem; S-WD increased the N and P concentrations of leaves and the N concentration of the pods. Moreover, all WD treatments retarded plant senescence. The WD revealed itself to be promising as a growth promoter for grain legumes, but further research is needed to understand the interference between the combined soil and foliar applications.

## 1. Introduction

The application in agricultural and environmental contexts of new materials derived from woody biomass has become a rising concern recently because of the benefits in terms of mitigating climate change and increasing carbon sequestration and soil fertility. Wood distillate (WD) is a dark brown/yellowish liquid with a typical smoky–sweetish odor that derives from the condensation of pyrolysis gases obtained when producing biochar from wood biomass under high temperature and hypoxia. It is composed primarily of water and more than 200 organic compounds of different types and quantities depending on the chemical composition and humidity of the feedstock biomass and on the pyrolysis conditions [1]. The composition in organic substances includes organic acids, ketones, aldehydes, alcohols, benzene and its derivatives, heterocyclic compounds, phenols and their derivatives, alkyl phenyl ethers, carbohydrate derivatives, and nitrogen compounds [2]. For the high content in acetic acid, the pH of WD is always acidic or very acidic.

For agricultural use, WD is classified as a corroborant, i.e., a natural substance which promotes plant growth, improves the resistance to pathogens and parasites, and also protects plants from damage not caused by parasites (Italian regulation, DPR 6793 of 18 July 2018) [3]. Moreover, unlike most fertilizers and pesticides, WD is not potentially dangerous for human health and for the environment [1]. At present, research on the application of WD in agriculture has just begun, and the most tested crops are rice, wheat, tobacco, and rapeseed. In rice, WD diluted 300 times increased yield and protein content, improved rice quality, and significantly enhanced photosynthesis and panicle number [4]. In wheat, seed soaking with WD diluted 600 times promoted germination and seedling growth, increased plant dry weight, and enhanced the tolerance to drought stress [5]. Spraying tobacco with a 300-fold dilution of WD significantly increased the yield and soluble protein and potassium content, and it enhanced antioxidant enzyme activity [6]. A 400-fold dilution of WD sprayed on rapeseed increased the primary leaf area and the number of pods per plant, but also increased seed yield and quality, and the resistance to pathogen fungi [7]. Due to the high content of polyphenolic compounds with antioxidant properties, WD was also found to protect lettuce plants from ozone injuries [8]. The application of WD to soil was found to enhance the availability of nutrients, as demonstrated by the increased concentration of nitrogen both in the topsoil and in wastewaters, probably through the stimulation of soil urease [9].

Field bean (*Vicia faba* var. *minor* Beck) is a forage legume which adapts well to a variety of soil and climate conditions and is used for grain, forage, and silage production throughout the northern hemisphere [10,11]. Its cultivation is promoted in Europe in order to diversify agroecosystems and increase the associated biodiversity [12]. Due to its ability to fix atmospheric nitrogen, the field bean is generally grown without N fertilization. However, because of the long flowering period, the maturing pods, which assimilate at the maximum efficiency for seed-filling, compete with immature pods and flowers, which both use assimilates at a lower rate [13]. Consequently, a large proportion of flowers and young pods are abscised after flowering, which reduces plant efficiency in the use of resources and is a primary reason for seed yield variability [14]. Salon et al. [15] found that the abortion of young reproductive structures relies primary on N shortage within the plant, also because N_2_ fixation generally declines after flowering due to the lower redirection of photosynthates to nodules [16]. To sustain seed-filling, N is remobilized from vegetative organs, thus fastening senescence, and causing the abortion of younger structures [15].

The supply of N as urea during grain filling could alleviate N limitations without compromising the N_2_ fixation capability of grain legumes [17], and reduced flower and pod abortion were found in field bean, soybean, and chickpea in response to N fertilization [18,19,20]. Pampana et al. [21] reported that, in the field bean, the supply of mineral nitrogen reduced the number of nodules, but different from other grain legumes, it increased the N_2_ fixation per unit of nodule biomass, demonstrating that *Rhizobium leguminosarum* bv. *viciae* was more tolerant to mineral N than other strains. On the other hand, Duc [10] reported that, more frequently, fertilization is responsible for flower abortion, as it favors vegetative structures in the competition for nutrients. It has been hypothesized that a slow-release N source might attenuate these negative effects of N fertilization, still making supplementary N available during the seed-filling period [22]. Pampana et al. [23] found that field beans fertilized with biosolids derived from sewage sludge produced a higher seed yield, which they attributed to the removal of the imbalance between the N demand and N supply during the reproductive stage. We hypothesize that similar benefits could be obtained by supplying WD.

Moreover, the field bean is susceptible to several pathogen fungi (*Botrytis fabae*, *Ascochyta fabae*, and *Uromyces viciae-fabae*), which attack the aerial part and primary leaves, while the roots may rot when attacked by *Fusarium* species [10]. Among pests, aphids pose the most serious problem, as they directly damage the plant by feeding on the phloem and are vectors of viruses [24]. In organic farming, narrow crop densities are proposed to control aphids, viruses, and weeds without using pesticides and herbicides [24,25]. On the other hand, high plant density may impair pollinator insects reaching the basal flowers, thus reducing their fertilization, and may also favor fungal infestations [24].

The use of natural products such as WD to protect the field bean and other legume crops from fungal diseases and other pests would be a promising alternative to xenobiotic products in conventional agriculture and a necessary aid in organic farming. The antifungal properties of WD were reviewed by Tiilikkala et al. [26], and, in a laboratory experiment, WD proved to be effective against the wood-decay fungi responsible for brown and white rot [27]. Moreover, WD is traditionally used as insect repellent in many populations [26]. As in the field bean, pollinator insects substantially increase the number of mature pods per plant, as well as the number of seeds in the pod, so it is crucial to assess if plant spraying with WD could alter the attractiveness of flowers [28,29]. Indeed, the strong smoky smell of WD could mask the flower scent, which is rewarded as an important trait influencing how likely a pollinator is to visit the flower.

To the best of our knowledge, the effects of wood distillate on both the chemical and biological properties of soil and the growth and reproductive performance of nitrogen-fixing plants have not been investigated together before. Therefore, we studied the influence of foliar, soil, and combined soil–foliar application of WD on field-bean (*Vicia faba* var. *minor* Beck) plants grown in the field on a sandy loam soil. A preliminary pot experiment was performed to elucidate the effects of soil application of WD on root and nodule growth. It is hypothesized that WD application to soil improves the availability of N and other nutrients to plants without impairing nodulation, and that WD aerial spraying ameliorates plant health without impairing the pod set.

## 2. Results

### 2.1. Effect on Soil Quality and Nutrient Content

The application of WD did not cause any variation in the pH and total organic carbon; the values were 8.2 for pH and 10.9 g kg^−1^ dry weight for TOC, averaged over all treatments.

Although WD-based treatments did not substantially influence the TOC content, the DOC and DOC fraction ratio were changed depending on the type of WD application applied (Table 1 and Supplementary Table S1). Indeed, if WD was applied as a foliar spray, DOC and DOC/TOC were lower than control (F-0, S-0) by approximately 5%, whereas the same parameters were higher than the control by approximately 7%, where WD was applied only to soil.

The soil biomass was also affected by the WD soil treatment, with an increase of about 9.7% for MB-C and about 8.2% for MB-C/TOC, but only when WD was applied to soil alone (Table 2 and Supplementary Table S2).

All types of WD applications, namely the foliar spray, soil irrigation, or combination, increased the tested soil enzymatic activities compared to the controls; however, the differences were significant only when it was applied to soil only (Table 3 and Supplementary Table S3). Indeed, the S-WD treatment alone increased the enzymes by using p-nitrophenol, TTF, and urea as substrates significantly compared to the controls: phosphatase (+17%), β-glucosidase (+27%), dehydrogenase (+45%), and urease (+50%). As a consequence of the increase in enzyme activities, the SAI3 was enhanced by the S-WD treatment by approximately 14% (Table 4 and Supplementary Table S4).

Opposite to the trends observed for the soil C fractions and the enzymatic activities, the soil irrigation with WD increased the concentration of bioavailable N and P both alone (S-WD) and in combination with foliar spray (FS-WD) (Table 5). For soil NO_3_^−^-N, the increase was by approximately 60%, whereas the concentration of available P was approximately 51% higher than in controls with the S-WD treatment, but only 18% higher with the combined application.

### 2.2. Effects on Field Bean Plants

**Figure 1:**
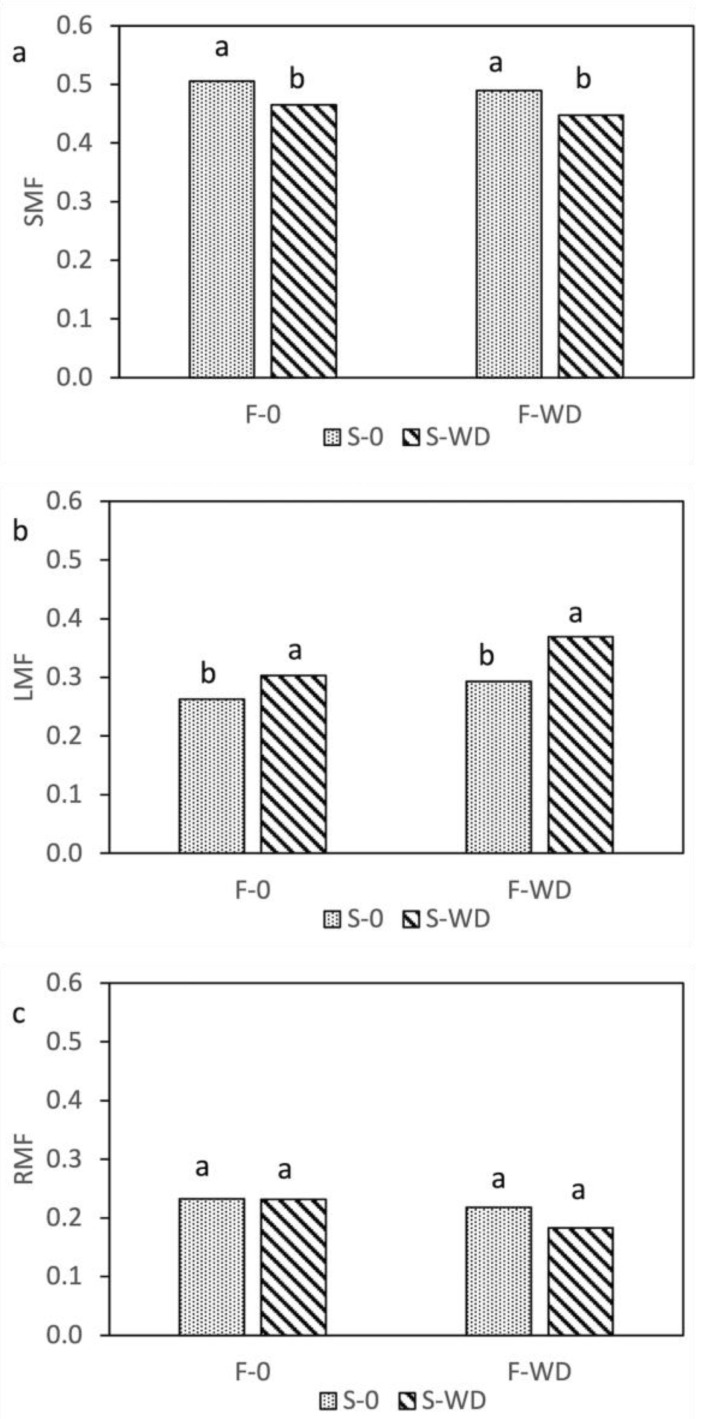
Stem ((**a**), SMF), leaf ((**b**), LMF), and reproductive ((**c**), RMF) mass fractions of field bean, as affected by the interaction of wood distillate (WD) distribution as foliar spray (F) or soil irrigation (S). Data are means of three replicates. For each parameter, columns with the same letter indicate not significantly different means at *p* ≤ 0.05, Tukey test.

The distribution of WD as foliar spray, soil irrigation, or combined did not significantly affect the biomass of stems, leaves, and reproductive structures (flowers and pods) of the field bean, and the shoot biomass was 1251 g m^−2^, averaged over all treatments. However, the partitioning of biomass within aboveground organs was influenced by WD, in that the leaf mass fraction was markedly higher, and correspondingly the biomass allocated into stems was lower, when WD was applied to soil with irrigation (Figure 1a,b and Supplementary Table S8). When WD was applied both to the leaves and to the soil, the proportion of shoot biomass allocated into reproductive structures was lower than in the other treatments (Figure 1c). Conversely, the control plants showed the highest stem and reproductive mass fractions and the lowest leaf mass fraction, which corresponded to the visual impression of a more advanced phenological stage of plants with an evident start of leaf senescence.

The chemical analyses highlighted that, in field bean plants, the N concentration was increased by approximately 10%: in leaves, this was with all WD applications, while in pods, it was only with soil irrigation (Table 5 and Supplementary Table S5). The P concentration of leaves was 15% higher than in controls with S-WD, whereas the P concentration of pods showed opposite trends, being 19% higher in the control plants. The concentrations of N and P measured in WD-treated plants compared to the controls support the visual impression that ripening was slightly delayed in response to WD, which could also explain the lower P concentration in the pods.

**Figure 2:**
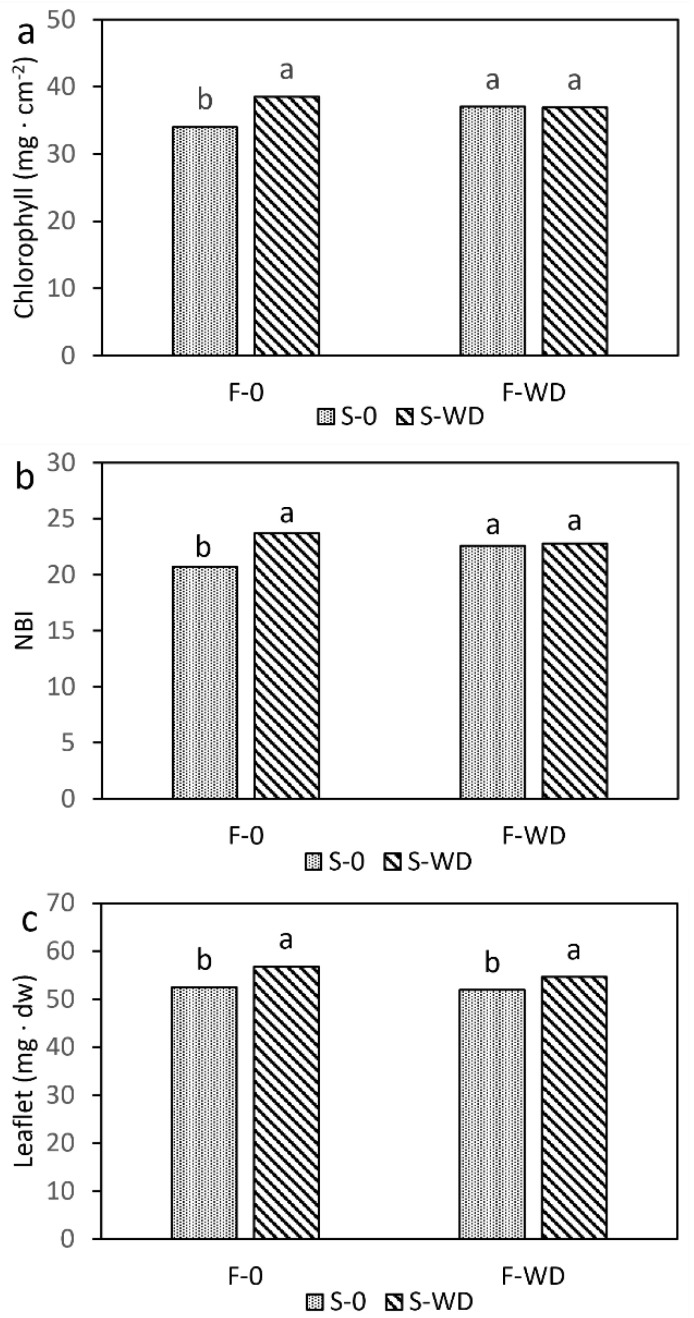
Chlorophyll concentration (**a**); nitrogen balance index, NBI (**b**); and biomass of field bean leaflets (**c**), as affected by the interaction of wood distillate (WD) distribution as foliar spray (F) or soil irrigation (S). Data are means of 20 replicates. For each parameter, columns with the same letter indicate not significantly different means at *p* ≤ 0.05, Tukey test.

In addition, the chlorophyll concentration per unit leaf surface and the nitrogen balance index were also significantly lower in the leaflets of the control plants (Figure 2a,b and Supplementary Table S9). The average dry weight of leaflets was slightly higher in WD-irrigated plants (Figure 2c), while the other leaflet parameters were not affected. Averaged over treatments, the number of leaflets per leaf was 6.2, the mean leaflet area was 13.5 cm^2^, and the surface leaf area was 25 mm^2^ mg^−1^.

The distribution of WD as foliar spray increased the number of fertile nodes and pods per stem, and this occurred independently of the distribution of WD to soil or not (Table 6). This result was not associated with earlier flower set or enhanced stem elongation, as the ranking of the most basal node bearing either flowers or pods was not affected, and stem height was even reduced by approximately 5.5 cm. The greater fertility of field-bean stems sprayed with WD did not lead to higher pod biomass per unit surface, because of the smaller pods at the time of harvest (Table 6 and Supplementary Table S6).

### 2.3. Effects on Roots

The results of the pot experiment confirmed that the irrigation with WD diluted 0.3% did not significantly affect the biomass of field bean shoots, and the same was recorded for roots (Table 7 and Supplementary Table S7). The number of nodules per plant did not change in response to the WD, while their weight was slightly but not significantly lower. In addition, the number and weight of nodules per unit root biomass was slightly higher when WD was distributed, thus highlighting that root growth was more affected than nodule initiation and growth. As a whole, the WD applied to soil only slightly reduced the root-to-shoot ratio, and this denotes that nutrient availability was not limiting.

## 3. Discussion

Our results demonstrated that the distribution of wood distillate both as a foliar spray and for soil irrigation improved the soil biological activity and the performance of field-bean plants. However, some conflicting effects were observed, especially when the two applications were combined.

The amount of dissolved organic carbon (DOC) of soil and its proportion on the total organic carbon (DOC/TOC%) were significantly higher in the soils irrigated with WD, whereas both parameters were lower when WD was applied to leaves. As DOC is composed mainly of organic acids and soluble carbohydrates [30], the former result was to be expected, due to the abundant presence of phenols and other organic substances in WD [31,32]. More critical is the interpretation of the negative effect of WD foliar spray on DOC and DOC/TOC%, and we hypothesize that it could depend on different sink–source relations within plants. At harvest, the leaf-sprayed plants showed, indeed, a higher number of pods per stem compared to the unsprayed plants, which probably exerted a strong sink for photosynthates and other organic substances, consequently reducing those redirected to roots and nodules [16] and, probably, also reducing the release of root exudates, such as easily decomposable polysaccharides, into the soil DOC pool. Unfortunately, we did not investigate the response of roots and nodules to leaf spray, and thus we have no data to confirm this hypothesis.

The easily available carbon forms present in WD, associated with the stimulating effect of phenols [33,34,35], enhanced the biomass of soil microbiota in the soil irrigation treatment. In the literature, significant increments of the soil bacteria community were reported with concentrations of WD comprised between 0.3% [36] and 2% [37,38], without changes in the microbial composition [36]. Moreover, Cardelli et al. [38] found that the application of WD did not accelerate the decomposition of the native organic matter, thus helping the storage of organic C in the soil (carbon sink). Similar to our findings, Liang et al. [39] reported that the increase in microbial biomass was also related to an increase in labile organic C, such as DOC. In our study, however, it is worth noting that the stimulating effect on soil microorganisms and biomass C obtained with WD irrigation was nullified when WD was applied also as foliar spray. In addition, all the tested enzyme activities were lowest in controls and highest with WD soil irrigation, while intermediate values were recorded when WD was applied as foliar spray, either in combination with soil irrigation or not. As a result, SAI3, which is an important parameter reflecting the soil quality based on the activities of the soil enzymes β-glucosidase, phosphatase, and urease [40], displayed the most negative score with the distribution of WD only to soil. SAI3 ranges from negative to positive scores without target values [41], and it is widely reported that negative SAI3 scores correspond to higher soil quality [42,43,44]. Moreover, Meyer et al. [39] reported that SAI3 correlates well with different tests and proved to be sensitive for discriminating among treatments and evaluating soil organic matter content and yield performance. The patterns of SAI3, of the tested enzyme activities, and of the microbial biomass C, found in our study suggest that the distribution of WD by soil irrigation improved the growing environment for the root system, but only when WD was not applied as foliar spray. We were not able to give insight into the mechanisms by which the foliar application of WD may have interfered with the stimulating effect on the soil biological activity exerted by WD irrigation. As for the patterns observed in DOC, we hypothesize that it could be related to differences in the plant status—maybe to a lower root decay—causing a reduced release of organic substrates supporting microorganism growth. Indeed, field-bean plants exposed to the combined WD treatments showed higher partitioning of biomass into leaves and lower into pods compared to only leaf-sprayed plants, despite the fact that the pod number and size were not affected, which, coupled to the retarded leaf senescence recorded in all WD treatments, suggest that plants were at a more juvenile growth stage.

Differences in plant status and sink–source relations largely rely on nutrient availability [10], and our research demonstrated that the distribution of WD through irrigation increased the concentration of NO_3_^−^-N, and available P in soil, while the foliar spray did not affect these parameters. An increase of available P in response to WD irrigation was reported for soils with both poor and medium available P content, and it was imputed to the ability of WD to decrease the soil pH [45,46]. As changes in soil pH were not recorded in our study, we attribute the higher P availability to the stimulation of phosphatase activity. Otherwise, the higher concentration of NO_3_^−^-N could be due to the proliferation of microorganisms, such as ammonia- and nitrite-oxidizing bacteria, involved in the nitrification process, which was also observed by adding WD to activated wastewater sludge [47].

The higher concentrations of N and P available forms in soil corresponded to a higher concentration of N in pods and of P in leaves. A similar response was found in *Ocimum basilicum* leaves [46], thus confirming the possible contribution of WD to realize a suitable soil environment for plant growth [48,49]. The opposite trend was observed for the P concentration in pods, as it was higher in controls than in WD treatments; this could be related to the more advanced ripening stage and could derive from the accumulation in pods of P remobilized from senescent leaves. Finally, the N concentration of leaves, the chlorophyll concentration, and the NBI were higher than in the controls with all types of WD distribution, thus indicating that WD components improved the nutrient balance of field bean plants also when distributed as a foliar spray, thus lengthening leaf duration. Moreover, Fedeli et al. [50] found that spraying lettuce with WD increased the concentration of chlorophyll, starch, and soluble sugars, which are all indices of increased photosynthetic performance. As it is known that polyphenols protect cells from oxidative stress both in humans and plants [51], we hypothesize that the polyphenol-rich WD could contribute to reduce oxidative stress and, thus, reduce the resort to photorespiration [52,53]. This would allow plants to maintain a higher photosynthetic efficiency in the later part of the growing season, which is characterized by high radiation and temperature in the Mediterranean area. To support this explanation, the diffusion of polyphenols through stomata should be assumed [54]. As a confirmation, Zhu et al. [7] reported that spraying rapeseed leaves with WD alone had a greater effect on delaying leaf senescence than when mixed with other growth regulators, such as gibberellin, sodium D-gluconate, and melatonin.

Both in the field and the pot experiment, the distribution of WD, either as a foliar spray or through soil irrigation, did not affect the field beans’ biomass, and it only slightly changed biomass partitioning within organs. The pot experiment revealed that the WD application to soil slightly reduced the allocation of resources to roots compared to the shoot, which is a general response to good nutrient availability in soil. Nevertheless, it also demonstrated that nodule initiation and growth were not inhibited by WD, suggesting that the changes in soil microorganisms and the release of NO_3_^−^-N into soil did not impair rhizobia infection but could support biological fixation during the reproductive phase [55].

According to Duc et al. [10], in legumes with an indeterminate growth habit such as that of our field bean, mineral N availability in soil favors vegetative growth, and this, in turn, promotes flower abortion. We found, indeed, that the pod number was not affected by the WD application to soil and was even increased by the foliar application, to which corresponded a reduced stem height. We explain the lower stem height in terms of increased competition for resources, in that the higher number of pod sinks caused a shortage of assimilates in the vegetative apex. It may be that the contrasting effect of soil and leaf applications of WD on field bean plants, i.e., increased mineral N availability prompting vegetative growth (S-WD), and higher pods per stem competing with vegetative growth (F-WD) are, at least in part, responsible for the conflicting results obtained with the combined application and reported in the literature [55]. Indeed, in rapeseed, in which stem and leaf growth do not compete with pod development, WD foliar spray increased the pod number without affecting plant height [7].

We did not analyze the incidence of pollinators and pests in this study, and therefore we cannot explain the way in which the WD promoted the pod number. However, we found that WD did not advance the reproductive phase, as the ranking of the first fertile node did not change significantly, while it reduced flower abortion, as more nodes were above the first fertile produced pods, despite the shorter plant height. We harvested plants before maturity, and therefore we can only speculate that the higher number of pods per stem recorded in plants receiving WD as foliar spray would lead to a higher seed yield. However, De Costa et al. [14] found a linear relationship between pod yield and leaf area duration, and the seed yield was correlated with the number of pod-bearing nodes. Moreover, Fedeli et al. [56] reported that chickpeas receiving WD as foliar spray over the entire growth cycle did not change the vegetative biomass compared to untreated plants but increased the mean weight and nutritional quality of seed, thus demonstrating a positive effect on the seed-filling process.

## 4. Materials and Methods

### 4.1. Field Experiment

#### 4.1.1. Experimental Setup

**Figure 3:**
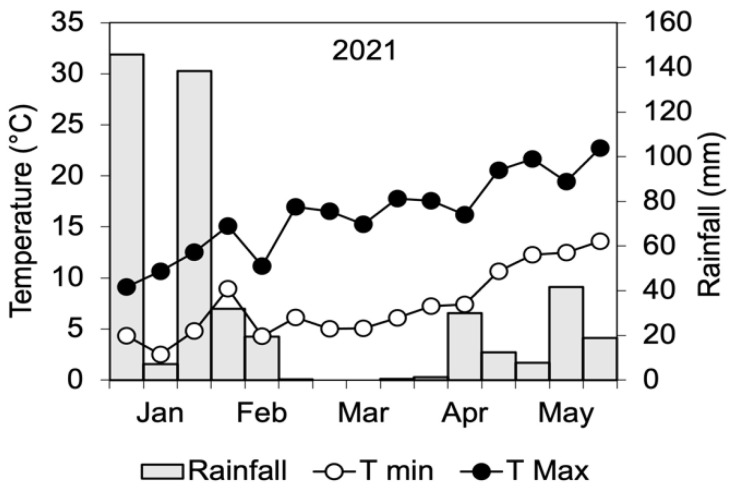
Decadic mean and maximum temperatures and cumulated rainfall during the period of wood distillate distribution to the field bean crop.

The research was carried out in 2021 at the “Rottaia” experimental station of the Department of Agriculture, Food and Environment (University of Pisa, Pisa, Italy). The experimental station is located approximately 3 km from the sea (43°40′34″ N, 10°18′41″ E) and 0 m a.s.l. The climate is hot-summer Mediterranean (Csa) according to the classification of Köppen. Over the entire period of the research, daily minimum and maximum temperatures and rainfall were obtained from a meteorological station located close to the trial site (Figure 3).

*Vicia faba* var. *minor* Beck, cv. Enrico (field bean), was sown on November 11th, spaced 15 cm apart (150 viable seeds m^−2^) on a field previously cultivated with *Avena sativa*. After the oat harvest, 20 cm deep soil tillage was performed with a disk harrow in July, followed by 40 cm deep soil ploughing after the brake of summer drought and by 10 cm deep soil harrowing twice with a rotary power harrow before sowing. Except for wood distillate (WD), no fertilizer was distributed throughout the cultivation period.

Wood distillate was produced by Bio-Esperia srl. (Arezzo, Italy) and was obtained from native forest plant species (*Abies* sp., *Alnus* sp., *Castanea sativa*, *Fraxinus* sp., *Quercus* sp., and *Robinia pseudoacacia*) through pyro-gasification. The distillation process was characterized by an average temperature of 50–70 °C hour^−1^ for 10 h, with a final peak of 1200 °C for about half an hour. The main characteristics of WD used in this study were as follows: pH, 2.8; density, 1.037 g mL^−1^; TOC, 33.8 g L^−1^; total N, 0.43 g L^−1^; organic acid, 32.3 g kg^−1^; phenolic compounds, 13.0 g L^−1^; and methanol, 13.4 g L^−1^. Polychlorobiphenyls and polycyclic aromatic hydrocarbons (PAHs) were determined by using solid-phase microextraction prior to their analysis by gas chromatography coupled to tandem mass spectrometry, but none of these toxic compounds was present in relevant concentrations. Among PAHs, only acenaphthylene and phenanthrene reached 0.09 ng L^−1^, well below the most restrictive legislative limits.

The treatment with WD started on February 12th, when field bean plants were at the leaf stage, with 3–5 unfolded leaves (13–15 BBCH [57]). Treatments were arranged in a split-plot experimental design, with WD distributed as foliar spray serving as the main plot and WD soil irrigation as the subplot, with three replicates. The four resulting treatments were the control (FS-0), without any WD distribution; only foliar spray (F-WD); only soil irrigation (S-WD); and combined aerial and soil irrigation (FS-WD). The size of each treatment plot was 5 m × 5 m, and a 1 m wide strip was cut to separate them. Wood distillate was distributed to all treatments at 10-day intervals 10 times (last time on May 13th). At each time, WD was applied to the S-WD and FS-WD plots as soil surface irrigation in the amount of 1 L m^−2^ (total 25 L) at the concentration of 0.3%, while the plants of the F-WD and FS-WD plots were sprinkled with 4 L of solution at 0.2% of WD. To equilibrate the water supply, the soil of the control plots and F-WD plots was irrigated with 25 L of water, while the control and S-WD plants were sprinkled with 4 L of water. Rainfall was very scarce during the treatment period (Figure 3), so that washout and dilution in soil could be excluded.

#### 4.1.2. Plant Harvest and Measurements

Plants were harvested at the stage of full flowering (May 18th), when all pods were still green and only basal ones had reached their final length (65–73 BBCH). Replicates consisted of the field bean plants growing in a 1 m row. Rows were all chosen with a stem number equal to 10 in order to avoid variability in density, as it could influence plant height, flower distribution, and flower fertilization. Plants were manually cut at ground level. For each stem, we recorded the following: height; the ranking of the most basal flower or pod bearing node, which was named 1st fertile node; the number of pod-bearing nodes; and the total number of pods longer than 1.5 cm. Plants were then separated into stems, leaves, and reproductive structures, which comprised both flowers and pods at all developmental stages. To determine the dry biomass, separate plant parts were oven dried at 65 °C to constant weight.

Total nitrogen and total phosphorus contents of leaves and pods were measured through the Kjeldahl and Olsen methods, respectively, adapted for vegetal tissues [46].

#### 4.1.3. Soil Sampling and Analysis

At plant harvest, soil samples were collected at a depth of between 0 and 20 cm. The soil was classified as sandy loam, Typic Xerorthent (USDA Soil Taxonomy). The main soil characteristics were analyzed by standard methods [58]: 67% sand, 22% silt, 11% clay, 45% water holding capacity (WHC), 8.2 pH, 1.09% organic C, 1.38 g kg^−1^ total N, 11 mg kg^−1^ available P, 79 mg kg^−1^ exchangeable K, and 10.4 cmol^(+)^ kg^−1^ cation-exchange capacity. Nitrates were extracted from soil by stirring it with distilled water (soil:H_2_O 1:5) for 1 h. Then the extract was filtered on a Whatman No. 42 paper disc and analyzed for NO_3_^−^-N by ion chromatography (Dionex DX120).

The total organic carbon (TOC) was determined by dry combustion (induction furnace 900 CS, Eltra, Haan, Germany) after removing carbonate carbon. Dissolved organic carbon (DOC) was determined by stirring soil samples with distilled water (soil/H_2_O 1:20) for 24 h at room temperature, centrifuging the suspension at 10,000× *g* rpm for 10 min, and, after filtration through a 0.45 mm glass fiber, determining the carbon with an OC analyzer for liquid samples (HachQbD1200). Soil microbial biomass carbon (MB-C) was determined according to Vance et al. [59] with the extraction of OC from fumigated and unfumigated soils by 1 N K_2_SO_4_. The OC was then measured by using a QBD1200 Laboratory TOC Analyzer (Hach Company, USA). An extraction efficiency coefficient (Kc) of 0.45 was used to convert the difference in soluble carbon between the fumigated and unfumigated soils into MB-C.

Dehydrogenase (Deh) activity was determined by following Tabatabai [60], based on a colorimetric assay, as the 2,3,5-triphenylformazan produced by the microorganism from the reduction of 2,3,5-triphenyltetrazolium chloride. β-Glucosidase activity (β-glu) was determined by a colorimetric method, using 4-nitrophenyl-β-D glucopiranosyde as a substrate: soil samples were incubated at 37 °C for 60 min; the reaction product p-nitrophenol was detected at 410 nm [61]. Following Eivazi and Tabatabai [62], alkaline phosphatase (APase) activity was based on the hydrolysis of p-nitrophenyl phosphate added to the soil samples. This phosphate releases p-nitrophenol, which can be detected colorimetrically. Arylsulfatase activity was determined by a colorimetric method, using p-nitrophenyl sulfate as a substrate: soil samples were incubated at 37 °C for 1 h, and the reaction product (p-nitrophenol) was extracted by dilute alkali (CaCl_2_ 0.5 M and NaOH 0.5 M) and detected at 400 nm [63]. Urease activity was determined according to Kandeler and Gerber [64], based on the spectrophotometric measurement of the ammonia released after a 2 h incubation of soil samples with a urea substrate at 37 °C.

The soil alteration index three (SAI3) was used to evaluate the influence of the several treatments on the quality and alteration degree of the soil. As reported in Puglisi et al. [41], SAI3 was determined through the conversion of the enzyme activity data in the following relationship:

SAI3 = (7.87 × β − glucosidase) − (8.22 × phosphatase) − (0.49 × urease) (1)

where enzyme activities were expressed in micromoles of p-nitrophenol per gram of soil per hour (for β-glucosidase and phosphatase) and in micrograms of urea per gram of soil per hour (urease).

### 4.2. Pot Experiment

#### 4.2.1. Experimental Setup

The experiment was carried out in 2020 at the Department of Agriculture, Food and Environment (University of Pisa, Italy). Seeds of *Vicia faba* var. *minor* Beck, cv. Enrico, were inoculated with *Rhizobium leguminosarum* biovar. *viciae* and grown in 6 L pots made from polyvinyl chloride (PVC) (30 cm long and 16 cm in diameter) fitted with a perforated PVC base. Pots were filled with a sandy loam soil 57.6% sand (2–0.05 mm), 32.8% silt (0.05–0.002 mm), 9.6% clay (<0.002 mm), 8.0 pH, 1.0 g kg^−1^ total nitrogen (Kjeldahl method), 15.8 mg kg^−1^ available P (Olsen method), and 103 mg kg^−1^ available K (BaCl_2_-TEA method), and they had a 17.4 cmol^(+)^ kg^−1^ cation-exchange capacity. Pots were placed outdoors and regularly irrigated with drip irrigation up to the setup of treatments. Inoculated seeds were sown four per pot on February 24th and then reduced to two plants per pot when the first plants reached the 2-leaves-unfolded stage (BBCH code 12). Exceeding plants were carefully removed along with their root system, which allowed us to record the absence of nodules. Only the plants with two completely unfolded leaves showed small swellings, white inside, on the tap root, thus demonstrating that the infection was proceeding but biological N_2_ fixation was not active.

#### 4.2.2. Treatments

The distribution of WD started on March 30th, when most plants were at the stage 12 BBCH. Each pot received 500 mL of WD diluted at 0.3% in tap water weekly. Control pots received the same volume of tap water. The experimental design consisted of two treatments (S-0, control; and S-WD, wood distillate) with six replicates, each consisting of one pot with two plants. Six irrigations were performed, which corresponded to a distribution of 15 mL of concentrated WD per pot.

#### 4.2.3. Harvest and Measurements

Plants were harvested on June 1st, at the stage of flowering (BBCH code 63). The aerial part was cut at ground level and separated into stems, leaves, and reproductive structures. Roots were gently washed under a flow of tap water and then divided into tap root and laterals. Nodules were picked and counted. All plant parts were oven dried at 65 °C to constant weight.

### 4.3. Statistical Analysis

The results from the field experiment were analyzed with a two-way ANOVA to test the effects of foliar (F) and soil (S) application of WD, and their interaction (FS). Data were arranged in a split-plot, with F as the main plot and S as a subplot, with three replicates. The JMPsoftware (SAS Institute, Inc., Cary, NC, USA) was utilized, and the Tukey post hoc test was used to separate significantly different means at *p* ≤ 0.05. Means from the pot experiment were analyzed to determine the statistical significance of differences with Student’s *t*-test. All data were checked for normality and the homoscedasticity of variances.

## 5. Conclusions

In conclusion, our study highlighted that the analyzed soil and plant parameters were differently affected by WD in dependence of its application to soil or leaves, and also that the application to leaves had a slight antagonist effect on the stimulation of enzyme activity exerted by the application to soil. Though preliminary, because it was obtained from a one-year field experiment and thus needs to be confirmed for other soil and weather conditions, this knowledge is of interest for developing further research assessing the distribution technique which is most appropriate for the expected result.

The advantages of soil distribution could be resumed as a direct effect on microbial activity, which, in turn, promoted field bean growth because of the increased availability of nitrate and phosphorus. Moreover, neither WD nor the higher nitrate in soil seemed to alter rhizobia infection. The advantages of foliar spray resulted in higher pod production, and all applications retarded leaf senescence. The higher number of pods per stem could be the consequence of higher flower production, pollination, or pod set, but the present research was not able to elucidate this point.

Compared to other growth promoters, such as hormones, WD has the advantage of being easily obtained with the same pyro-gasification process used for the production of gas and biochar. Woody residues deriving from forest operations and manufacturing processes can be used as starting materials, thus promoting both recycling and circular economy. Due to the variable nature of the original woody feedstocks, WD is a complex mixture of organic compounds, and therefore it is expected to have a broad spectrum of possible positive and negative effects on biological activities, which can also vary in dependence on plant species and environmental conditions. In comparison, products with a single active substance represent a simpler construct in which the physiological effects and mechanism of action can be more easily determined. However, the effectiveness of WD-like complex biostimulants has been proved in practice, without understanding the mechanisms or mode of action yet, and, for their use, it is sufficient to have developed production processes that guarantee the uniformity of product performance over time [65].

Due to the compound positive effects on soil biology and crop growth, our results suggest that WD could be profitably used for sustainable crop management. However, further multi-year agronomic experimentation, coupled to molecular and biochemical investigations, is undoubtedly needed to identify the active components in the extracts and to establish their mechanisms of action on soil and plant processes.

## Figures and Tables

**Table 1:** Amount of soil dissolved organic carbon (DOC) and the DOC/TOC ratio as affected by the mean effects of foliar spray (F) and soil irrigation (S) with wood distillate (WD). Data are means of two treatments and three replicates (*n* = 6). For each treatment and parameter, same letters indicate not significantly different means at *p* ≤ 0.05, Tukey test.

Treatments	DOC (mg kg^−1^)	DOC (% TOC)
Foliar spray		
F-0	192.5 a	1.19 a
F-WD	123.2 b	1.13 b
Soil irrigation		
S-0	122.3 b	1.12 b
S-WD	130.4 a	1.20 a

**Table 2:** Amount of soil biomass carbon (MB-C) and the MB-C/TOC ratio as affected by the interaction of wood distillate (WD) distribution as foliar spray (F) and soil irrigation (S). Data are means of three replicates (*n* = 3). For each parameter, same letters indicate not significantly different means at *p* ≤ 0.05, Tukey test.

Treatments	MB-C (mg kg^−1^)		MB-C/TOC (%)	
	S-0	S-WD	S-0	S-WD
F-0	144 b	158 a	1.34 b	1.45 a
F-WD	143 b	139 b	1.32 b	1.33 b

**Table 3:** Soil enzyme activities as affected by the interaction of wood distillate (WD) distribution as foliar spray (F) and soil irrigation (S). APase, alkaline phosphatase; β—glu, β—glucosidase; Deh, dehydrogenase; Ure, urease. Data are means of three replicates (*n* = 3). For each enzyme activity, same letters indicate not significantly different means at *p* ≤ 0.05, Tukey test.

Treatments	S-0	S-WD	S-0	S-WD
	APase (µg p-nitrophenol g^−1^ h^−1^)		β—glu (µg p-nitrophenol g^−1^ h^−1^)	
F-0	321 b	376 a	51.0 b	69.7 a
F-WD	335 ab	338 ab	64.3 ab	63.0 ab
	Deh (µg TTF g^−1^ h^−1^)		Ure (µg NH_4_^+^-N g^−1^ 2 h^−1^)	
F-0	3.87 b	5.61 a	16 b	24 a
F-WD	4.82 ab	5.26 ab	20 ab	21 ab

**Table 4:** Soil alteration index (SAI3) as affected by the interaction of wood distillate (WD) distribution as foliar spray (F) and soil irrigation (S). Data are means of three replicates (*n* = 3). Same letters indicate not significantly different means at *p* ≤ 0.05, Tukey test.

Treatments	SAI3	
	S-0	S-WD
F-0	−16.4 b	−18.7 a
F-WD	−16.5 ab	−16.8 b

**Table 5:** Nitrogen and phosphorus concentration in the soil and in the leaves and pods of field bean, as affected by the interaction of wood distillate (WD) distribution as foliar spray (F) and soil irrigation (S). Data are means of three replicates. For each parameter, means followed by the same letter are not significantly different at *p* ≤ 0.05, Tukey test.

Treatment	Soil		Leaves		Pods	
	S-0	S-WD	S-0	S-WD	S-0	S-WD
	NO_3_^−^-N (mg kg^−1^)		N concentration (%)			
F-0	4.3 b	7.3 a	4.1 b	4.4 ab	3.7 b	4.1 a
F-WD	4.9 b	7.4 a	4.6 a	4.6 a	3.8 b	4.0 a
	Available P (mg kg^−1^)		P concentration (mg g^−1^)			
F-0	7.3 b	11.0 a	3.3 b	3.8 a	3.2 a	2.7 b
F-WD	7.9 b	8.6 ab	3.3 b	3.5 ab	2.7 b	2.7 b

**Table 6:** Biometric and reproductive traits of field-bean stems as affected by the application of wood distillate (WD) as foliar spray. Data are means of two soil treatments and three replicates (*n* = 6). For each parameter, means followed by the same letter are not significantly different at *p* ≤ 0.05, Tukey test.

Treatments	Stem Height	Fertile Nodes	1st Fertile Node	Pods		
	(cm)	(n stem^−1^)	(Node Ranking)	(n stem^−1^)	(mg pod^−1^)	(g m^−2^)
F-0	105.3 a	7.3 b	5.3 a	9.0 b	283.7 a	299.1 a
F-WD	95.8 b	9.0 a	4.6 a	12.1 a	189.9 b	237.6 b

**Table 7:** Shoot and root biomass and nodule traits of the field bean as affected by the application of wood distillate (WD) as soil irrigation in the pot experiment. Data are means ± SE of six replicates (*n* = 6).

Treatments	Shoot (g plant^−1^)	Roots (g plant^−1^)	Root/Shoot Ratio	Nodule Number (n plant^−1^)	Nodule Mass (mg plant^−1^)	Nod. Density (n g root^−1^)	Nod./Root (mg g^−1^)
S-0	3.9 ± 0.27	2.1 ± 0.31	0.54 ± 0.07	170 ± 15.4	103 ± 19.4	81.2 ± 4.9	49.4 ± 3.8
S-WD	4.0 ± 0.49	1.9 ± 0.20	0.47 ± 0.07	168 ± 22.9	96.3 ± 13.4	89.8 ± 9.6	51.4 ± 5.1

## Data Availability

The data presented in this study are available in the article or in the Supplementary Materials. The raw data supporting the conclusions of this manuscript are made available by the authors to any qualified researcher who makes a request.

## Supplementary Materials

The following supporting information can be downloaded at: https://www.mdpi.com/article/10.3390/plants12010121/s1, Table S1: Results of ANOVA Table 1; Table S2: Results of ANOVA Table 2; Table S3: Results of ANOVA Table 3; Table S4: Results of ANOVA Table 4; Table S5: Results of ANOVA Table 5; Table S6: Results of ANOVA Table 6; Table S7: Results of ANOVA Table 7; Table S8: Results of ANOVA Figure 1; Table S9: Results of ANOVA Figure 2.
