# Supplementary material for: Soil and Foliar Applications of Wood Distillate Differently Affect Soil Properties and Field Bean Traits in Preliminary Field Tests
Source: Plants (Basel). 2022 Dec 26;12(1):121. doi: 10.3390/plants12010121 (PMC9823333; doi:10.3390/plants12010121)
Supplement: Supplementary file 1 [file plants-12-00121-s001.zip › plants-2054792-supplementary.pdf]

Table S1. Results of ANOVA Table 1.

Source of Variation		DOC (mg kg^{-1})	DOC (% TOC)
Foliar	F	5.6411	40.50
	P > F	0.0449*	0.0002*
Soil	F	9.2271	392.00
	P > F	0.0161*	0.0001*
Foliar × Soil	F	3.8028	32.00
	P > F	0.0870	0.0005*

Table S2. Results of ANOVA Table 2.

Source of Variation		MB-C (mg kg^{-1})	MB-C/TOC (%)
Foliar	F	13.5941	7.5519
	P > F	0.0062*	0.0003*
Soil	F	13.5188	37.4899
	P > F	0.0097*	0.0251*
Foliar × Soil	F	10.8828	36.7069
	P > F	0.0109*	0.0003*

Table S3. Results of ANOVA Table 3.

Source of Variation		APase (μg p-nitrophenol g^{-1} h^{-1})	β – glu (μg p-nitrophenol g^{-1} h^{-1})	Deh (μg TTF g^{-1} h^{-1})	Ure (μg NH_4^+-N g^{-1} 2h^{-1})
Foliar	F	1.8413	0.8565	4.1971	2.1818
	P > F	0.2118	0.3818	0.0747	0.1779
Soil	F	8.3941	5.7901	53.7693	24.2424
	P > F	0.0200*	0.0428*	0.0001*	0.0012*
Foliar × Soil	F	9.9475	7.7088	19.5545	40.9697
	P > F	0.0135*	0.0241*	0.0022*	0.0002*

Table S4. Results of ANOVA Table 4.

Source of Variation		SAI3
Foliar	F	4.5557
	P > F	0.0653
Soil	F	8.9292
	P > F	0.0174*
Foliar × Soil	F	5.6039
	P > F	0.0454*

Table S5. Results of ANOVA Table 5.

Source of Variation		Soil		Leaves		Pods	
		NO_3^--N (mg kg^{-1})	Available P (mg kg^{-1})	N conc. (%)	P conc. (mg g^{-1})	N conc. (%)	P conc. (mg g^{-1})
**Foliar**	F	0.0426	4.0833	1.7933	2.7292	0.6108	8.2043
	P > F	0.8417	0.0780	0.2173	0.1371	0.4570	0.0210*
Soil	F	360.1702	14.0833	11.7933	12.2229	46.7656	10.3836
	P > F	0.0001*	0.0056*	0.0217*	0.0081*	0.0001*	0.0122*
Foliar × Soil	F	12.2979	6.7500	15.1766	9.1232	12.687	11.5694
	P > F	0.0082*	0.0317*	0.0462*	0.01152*	0.0095*	0.0093*

Table S6. Results of ANOVA Table 6.

Source of Variation		Stem Height	N° Fertile Nodes	1st Fertile Node	Pods (n stem^{-1})	Pod (mg)	Pods (g m^{-2})
Foliar	F	10.758	24.631	11.206	7.090	10.480	30.092
	P > F	0.0112*	0.011*	0.0101*	0.0287*	0.0119*	0.0006**
Soil	F	0.9570	3.5567	2.1879	0.0369	0.3115	0.8214
	P > F	0.3566	0.0960	0.1774	0.8524	0.5920	0.3912
Foliar × Soil	F	0.2682	0.2463	5.0970	0.0008	0.0498	1.5651
	P > F	0.6185	0.6330	0.0539	0.9788	0.8289	0.2463

Table S7. Results of Student test Table 7.

Source of Variation		Shoot (mg plant^{-1})	Root (mg plant^{-1})	R/S	Nodule (n plant^{-1})	Nodule (mg plant^{-1})	Nodule (n g root^{-1})	Nod/Root (mg g^{-1})
Soil	t	-0.25	0.53	0.38	0.05	0.23	-0.64	-0.59
	P > t	0.811	0.611	0.715	0.961	0.822	0.5386	0.5667

Table S8. Results of ANOVA Figure 1.

Source of Variation		SMF	LMF	RMF
Foliar	F	1.9756	4.9793	4.2000
	P > F	0.1975	0.0562	0.0746
Soil	F	15.244	6.3472	1.1524
	P > F	0.0045*	0.0358*	0.3144
Foliar × Soil	F	0.0244	0.6269	1.1524
	P > F	0.8798	0.4513	0.3144

Table S9. Results of ANOVA Figure 2 and other leaflet traits cited in the text.

Source of Variation		Chl	NBI	Leaflet (mg leaflet^{-1})	Leaflet (n leaf^{-1})	Leaflet (cm^2 leaflet^{-1})	SLA (mm^2 mg^{-1})
Foliar	F	1.6465	1.0469	0.2712	0.1030	0.0156	0.5408
	P > F	0.2033	0.3095	0.6041	0.7492	0.9010	0.4644
Soil	F	8.6525	9.9526	4.4624	0.0000	0.8950	0.3750
	P > F	0.0043*	0.0023*	0.0379*	1.0000	0.3471	0.5421
Foliar × Soil	F	8.1412	5.6996	0.6975	2.5745	0.0182	0.1121
	P > F	0.0056*	0.0195*	0.4063	0.1127	0.8931	0.7387
